# The molecular sociology of NHERF1 PDZ proteins controlling renal hormone-regulated phosphate transport

**DOI:** 10.1042/BSR20231380

**Published:** 2024-03-27

**Authors:** Peter A. Friedman, Tatyana Mamonova

**Affiliations:** 1 Laboratory for G Protein-Coupled Receptor Biology, Department of Pharmacology and Chemical Biology, University of Pittsburgh School of Medicine, Pittsburgh, PA 15261, U.S.A.; 2 Department of Structural Biology, University of Pittsburgh School of Medicine, Pittsburgh, PA 15261, U.S.A.

**Keywords:** FGF23 and PTH, NHERF1 and NPT2A, PDZ domain, PDZ-ligand interacation, phosphate transport, phosphorylation/dephosphorylation

## Abstract

Parathyroid hormone (PTH) and fibroblast growth factor-23 (FGF23) control extracellular phosphate levels by regulating renal NPT2A-mediated phosphate transport by a process requiring the PDZ scaffold protein NHERF1. NHERF1 possesses two PDZ domains, PDZ1 and PDZ2, with identical core-binding GYGF motifs explicitly recognizing distinct binding partners that play different and specific roles in hormone-regulated phosphate transport. The interaction of PDZ1 and the carboxy-terminal PDZ-binding motif of NPT2A (C-TRL) is required for basal phosphate transport. PDZ2 is a regulatory domain that scaffolds multiple biological targets, including kinases and phosphatases involved in FGF23 and PTH signaling. FGF23 and PTH trigger disassembly of the NHERF1–NPT2A complex through reversible hormone-stimulated phosphorylation with ensuing NPT2A sequestration, down-regulation, and cessation of phosphate absorption. In the absence of NHERF1–NPT2A interaction, inhibition of FGF23 or PTH signaling results in disordered phosphate homeostasis and phosphate wasting. Additional studies are crucial to elucidate how NHERF1 spatiotemporally coordinates cellular partners to regulate extracellular phosphate levels.

## Introduction

**Figure 1:**
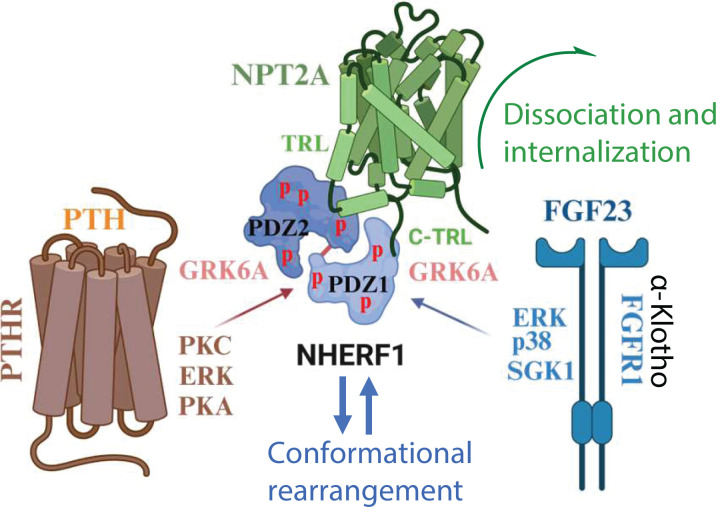
Regulation of the NHERF1–NPT2A complex by PTH and FGF23 The Na^+^-dependent phosphate cotransporter-2A (NPT2A) binds Na^+^/H^+^ Exchanger Regulatory Factor-1 (NHERF1) PDZ1 and PDZ2 through the carboxy-terminal (C-TRL) and internal (TRL) PDZ-binding motifs, respectively. The NHERF1–NPT2A complex is regulated by fibroblast growth factor-23 (FGF23) and parathyroid hormone (PTH). Parathyroid hormone receptor (PTHR)-PTH action (left) is mediated by PKC and PKA, whereas fibroblast growth factor receptor 1 (FGFR1)-FGF23 pathway (right) includes p38γ and SGK1. Activated kinases phosphorylate NHERF1 at multiple sites (p). Conformational changes promoted by phosphorylation of NHERF1 allow GRK6A to bind NHERF1, phosphorylate Ser^290^, disengage NPT2A, and arrest phosphate transport.

**Figure 2:**
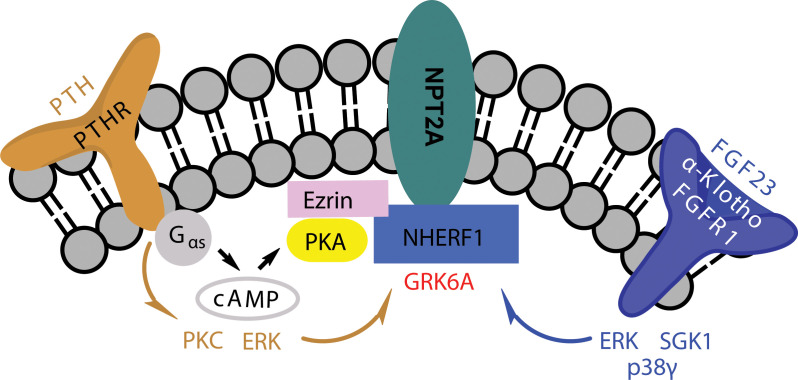
Working model for FGF23- and PTH-mediated inhibition of phosphate transport in human kidney cells NHERF1 binding to NPT2A stabilizes the NHERF1–NPT2A complex at the plasma membrane to facilitate renal phosphate transport. PTH acting through the PTHR-Gs-cAMP-PKA, PKC, or ERK pathways phosphorylates NHERF1 to dissociate from NPT2A and blocks phosphate uptake. Similarly, FGF23 acting through FGFR1c/α-Klotho, ERK, SGK1, or p38γ pathways phosphorylates NHERF1 to uncouple it from NPT2A and block phosphate uptake. Shared PTH and FGF23 activity involves ERK and GRK6A.

**Figure 3:**
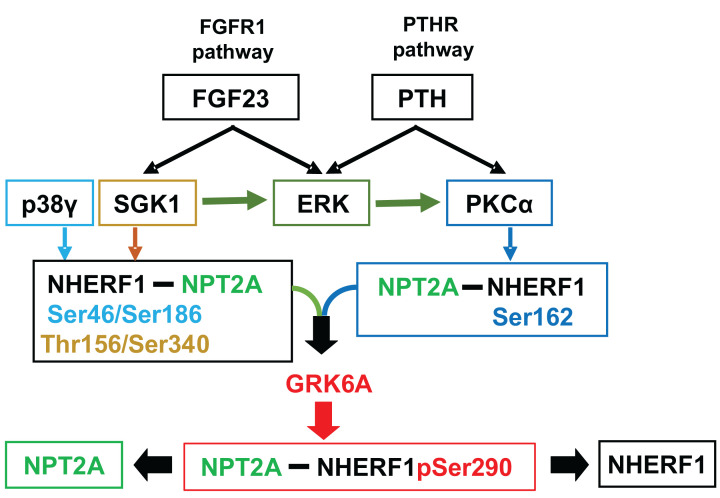
FGF23 and PTH pathways converge on NHERF1 phosphorylation FGF23- and PTH-stimulated p38γ, SGK1, ERK, and PKCα activity converge at GRK6A, promoting common Ser^290^ (pSer^290^) phosphorylation to regulate NPT2A-dependent phosphate uptake. The C-terminal motif of NPT2A interacts with NHERF1 PDZ1, whereas the C-terminal motif of p38γ, SGK1, PKCα, or GRK6A binds NHERF1 PDZ2 to phosphorylate Ser^46^/Ser^186^, Thr^156^/Ser^340^, Ser^162^, or Ser^290^.

Phosphate is continuously absorbed from the intestines and primarily stored in bone and teeth. Phosphate wasting or hypophosphatemia associated with malnourishment, chronic kidney disease, and frank resistance to hormone action contributes to exceptionally high mortality rates, especially among the elderly and impoverished [1–3]. Phosphate serum levels and homeostasis are achieved by parathyroid hormone (PTH) and fibroblast growth factor-23 (FGF23) acting through a bone-kidney axis [4]. PTH and FGF23 regulate NPT2A-mediated Na^+^-phosphate cotransport by a mechanism requiring NHERF1, a PDZ scaffold phosphoprotein [5–9]. In the presence of NHERF1, PTH works through its cognate G protein-coupled receptor (PTHR) [10] and FGF23 via a receptor tyrosine kinase (FGFR1) and α-Klotho [11] to block NPT2A-mediated phosphate uptake (Figure 1). PTHR and FGFR1 are members of structurally disparate receptor classes with distinct signaling pathways (Figure 2). Inexplicably, sometimes PTH and FGF23 act in concert but at other times independently [12–15]. The mechanism underlying these events is not understood. Nonetheless, operating through distinct kinases (Figure 1), both pathways converge to activate G protein-coupled receptor kinase 6A (GRK6A) [16–18]. GRK6A binds NHERF1 and phosphorylates it at Ser^290^, triggering dissociation of NPT2A from NHERF1 and terminating hormone-sensitive phosphate transport [17] (Figure 3). It is a biological riddle of how a GPCR and an FGFR phosphorylate the same obligate residue to regulate phosphate uptake without interfering with each other.

## NHERF1

NHERF1 (SLC9A3R1) is a multi-domain PDZ scaffolding protein expressed at proximal renal tubular apical membranes and in osteoblasts [19–21]. Mice lacking NHERF1 [22–24] and humans harboring mutations or polymorphisms in *SLC9A3R1*, who are haploinsufficient for NHERF1 [25,26], exhibit hypophosphatemia, osteopenia, and increased fracture rates. NHERF1 tethers binding partners through tandem PDZ domains named for the common structural domain shared by the postsynaptic density protein (PSD95), Drosophila disc large tumor suppressor (DlgA), and zonula occludens-1 protein (ZO-1), and the C-terminal ezrin-binding domain (EBD) associated with ezrin [27]. NHERF1 PDZ modules consist of 90 amino acids forming a 3D globular domain that is composed of six β-sheets (βA-βF) and two α-helices (αA and αB) [28]. NHERF1 PDZ1 and PDZ2 have similar sequences, including identical core-binding motifs (-GYGF-) essential for the interaction with a carboxy-terminal (C-terminal) linear fragment of ligand partners that are 3-to-4 residues in length (X-S/T-X-Φ_COO_^−^ class I PDZ-recognition motifs, where X is any amino acid and Φ is a hydrophobic residue) [28]. These residues are numbered starting from the terminal position (P^0^) and going backward to P^−1^, P^−2^, P^−3^, etc. Though NHERF1 PDZ domains are very similar, they exhibit distinct ligand specificity. While PDZ1 and PDZ2 domains both recognize the carboxy-terminal four-residue motifs of selected proteins, structural specificity determinants span the entire binding groove [29]. Salt bridges between Arg^40^ in the β3 strand of PDZ1 and Glu-3 in PTHR (-E^−3^TVM), Asp-3 in CFTR (-D^−3^TRL) or Asp-3 in β2-AR (-D^−3^SLL), as well as between Arg^180^ of the β3 strand of PDZ2 and Glu-3 in PTHR, significantly stabilize PDZ-ligand complexes. Thus, binding specificity derives from the core GYGF motif, upstream determinants in the target peptide, and 3D elements outside the PDZ-binding grooves.

NHERF1 PDZ1 interacts with the type-2 sodium-phosphate cotransporter NPT2A (*SLC34A1*) [30,31] via its C-terminal PDZ-ligand motif -Thr^−2^Arg^−1^Leu^0^ (C-TRL), wherein the interaction with PDZ2 is insignificant [27,32,33] despite the PDZ2 domain exhibits a very similar primary sequence with an identical core binding site through which it interacts with the target ligands [18,28,34–36].

Recently, it was identified that the transmembrane protein 174 (*Tmem174*) is significantly coexpressed with *Slc34a1*. Tmem174 is a kidney-specific protein located at the apical membrane of renal proximal tubular cells [37,38]. Furthermore, it was suggested that TMEM174 interacts with NPT2A, but not NHERF1, and regulates NPT2A by PTH and FGF23 in human kidney [37,38]. We speculate that TMEM174–NPT2A–NHERF1 may form a ternary complex at apical membranes of renal proximal tubules. A multistep mechanism would dissociate the complex in this scenario, permitting FGF23- and PTH-mediated NPT2A internalization.

The specificity of the interaction between NHERF1 PDZ1 and NPT2A relates to Glu^43^ and Arg^−1^ in PDZ1 and the NPT2A C-TR^−1^L, respectively [29,32,33]. Asp^183^, located at the homologous position in PDZ2, has a shorter side chain compared with Glu^43^ to form a direct interaction with Arg^−1^ [32]. PDZ2 with the Asp^183^Glu rescue mutation interacts with Arg^−1^, comparable with PDZ1, but does not support hormone-sensitive phosphate transport, therefore underlines the importance of the binding between PDZ1 and NPT2A for hormone regulation [32,33]. It should be noted that NPT2C, a *SLC34A3* paralog expressed in the kidney, lacks a PDZ ligand, does not bind NHERF1, and supports approximately 30% of Na^+^-dependent phosphate transport (Npt2a handles 70%). Overall PTH and FGF23 action on Npt2c is still unclear [39,40].

NHERF1 is a phosphoprotein possessing 31 Ser and 9 Thr residues. Although these sites are dispersed throughout the protein, a conspicuous Ser-rich cluster is located in the linker region between PDZ2 and the EBD. Phosphorylation is the most prevalent reversible posttranslational modification regulating NHERF1 activity and signaling [17]. Current understanding of renal phosphate transport suggests that the NPT2A–NHERF1 complex is down-regulated by PTH and FGF23 [15,41]. This model (Figures 1 and 2) assumes that PTHR and FGFR1 activate kinases that phosphorylate NHERF1 at specific sites required for their phosphaturic action [22,23,27,42,43]. Phosphorylation and the attendant NHERF1 conformational changes promote NPT2A dissociation from NHERF1. NPT2A is internalized extensively, whereas NHERF1 remains at the apical membrane [33]. The loss-of-function mutations or polymorphisms in *SLC9A3R1* (NHERF1) (E^68^A, L^110^V, R^153^Q, and E^225^K) [25,26] prevent assembly or disassembly of the NPT2A-NHERF1 complex and account for PTH and FGF23 resistance, which impedes down-regulation of NPT2A [5,15,36]. PTH signaling occurs through its cognate GPCR, PTHR [44–46]. PTHR is expressed at apical and basolateral membranes of proximal tubule cells [47,48]. At apical membranes, PTHR binds NHERF1 and regulates apical phosphate uptake by NPT2A [49,50]. The mechanism of membrane-delimited PTHR signaling via heterotrimeric Gsα/PKA and Gαq/PLC/PKC is largely characterized [8,10,45,46,51–55]. The PTH/PTHR signaling catalyzes the activity of adenylate cyclase via Gαs to convert ATP into cAMP`, the chemical messenger that activates PKA [8,10]. Inhibiting PKA blocks PTH-sensitive phosphate transport [8,31]. Gαq activates phospholipase Cβ (PLCβ), which in turn regulates second messengers diacylglycerol (DAG), inositol (1,4,5) -trisphosphate (IP3), releases Ca^2+^, and activates PKC [10] required to inhibit phosphate transport [52]. PTHR internalization to endosomes additionally elicits sustained cAMP formation [56,57]. Its role, if any, in regulating phosphate transport remains to be determined.

Similar to the PTH/PTHR action on NHERF1 [45,46,58,59], FGF23 working through a receptor tyrosine kinase (FGFR1) independently activates a signaling pathway that also impinges on NHERF1 [60–62]. FGF23 activates SGK1 [11,36,63] and MAP kinases [36,43]). The MAP kinase pathways are not entirely resolved, and substrate (NHERF1 or NPT2A) phosphorylation sites and their functional roles remain undefined. Activation of ERK1/2 by PTH and FGF23 is essential for inhibiting phosphate transport [36] and downstream activation of SGK1 in the case of FGF23 [63]. Despite two independent phosphorylation pathways stemming from PTHR and FGFR1 examined a decade ago, a complete understanding of how two pathways converge on and phosphorylate NHERF1 is absent (Figure 3) [17,18].

## NPT2A

**Figure 4:**
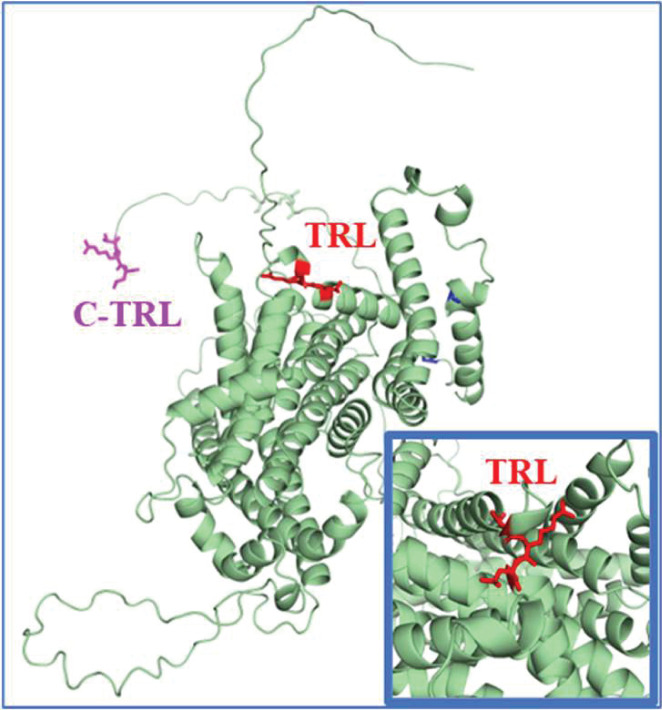
A computational model of NPT2A AlphaFold2 predicts the structure of NPT2A. Carboxy-terminal (C-TRL) and internal TRL PDZ motifs are depicted in magenta and red, respectively. The insert shows the orientation of the NPT2A internal TRL PDZ motif. Such an internal motif may establish a stable secondary structure sterically nestled in the binding groove of the PDZ domain.

The type-2 sodium-phosphate cotransporter (NPT2A, *SLC34A1*) is the principal protein mediating hormone-sensitive kidney phosphate absorption [64,65]. It is primarily expressed in kidney proximal tubules [66,67] and osteoblasts [68,69]. Mice lacking NPT2A display FGF23-independent renal phosphate wasting and hypercalciuria [67]. The predicted topology of NPT2A consists of 8 membrane-spanning segments and two helical hairpins [70,71]. AlphaFold [72] provided additional structural details of NPT2A (Figure 4). Retrieval from the cell membrane and sequestration controls NPT2A and related SLC34 proteins, unlike most Na^+^-coupled transporters whose function is regulated directly by posttranslational modifications [73]. FGF23 and PTH trigger endocytosis [30,42,65] but only in the presence of NHERF1 because the NPT2A C-TRL binds NHERF1 PDZ1, and this interaction establishes NPT2A apical localization [33]. In addition to its canonical carboxy-terminal PDZ ligand, NPT2A possesses a second previously uncharacterized internal PDZ ligand (-Thr^−2(494)^Arg^−1(495)^Leu^0(496)^) [33] (Figure 4). Such an internal motif may establish a stable secondary structure sterically nestled in the binding groove of the PDZ2 domain [74,75]. Loss-of-function mutations in this cryptic internal motif (Arg^495^His or Arg^495^Cys) that were not recognized as part of a PDZ ligand cause congenital phosphate wasting and hypophosphatemia [76,77]. Unlike WT NPT2A, neither Arg^495^Cys nor Arg^495^His dissociates from NHERF1 upon challenge with PTH [33] and fails to terminate phosphate transport. Concurrently, as demonstrated by confocal fluorescence microscopy, NPT2A Arg^495^Cys and Arg^495^His variants colocalized with NHERF1 at apical cell membranes like WT NPT2A [33]. Consistent with the functional results, NPT2A Arg^495^Cys and Arg^495^His variants do not internalize in response to PTH but remain at the apical membrane. Enigmatically, neither Arg^495^Cys nor Arg^495^His mutations are at the -1 locus (Arg^495^) of the PDZ ligand, considered a permissive position [78]. Notably, replacing Arg^495^ at the PDZ -1 position with disease-associated mutations Cys or His impairs Thr^494^ phosphorylation compared with WT NPT2A [33]. Using Alphafold2 and MD simulations, we found that Thr^494^ and Leu^496^ are solvent-exposed and may interact with NHERF1 [33]. Thr^494^ phosphorylation was detected in cells transfected with WT NHERF1 but not NHERF1 PDZ1, verifying the requirement for NHERF1 PDZ2 [33]. The latter may explain why extensive mutagenesis studies by Murer and colleagues failed to identify phosphorylation residues that accounted for the effect of PTH on the apical membrane abundance of Npt2a [79,80]. We advance a model wherein the carboxy-terminal NPT2A PDZ ligand binds NHERF1 PDZ1 and defines apical localization of cotransporter, while the internal NPT2A PDZ ligand controls hormone-triggered phosphate transport through the interaction with PDZ2 [33].

## PTHR

The parathyroid hormone receptor (PTHR), a Family B G-protein coupled receptor (GPCR), is a crucial regulator of mineral-ion metabolism and bone physiology [10,45,46]. NHERF1 is essential in receptor endocytosis and recycling [19,46,81,82]. PTHR binds to the PDZ domains of NHERF1 through its Class I type C-terminal PDZ-binding motif (-Glu^−3^Thr^−2^Val^−1^Met^0(593)^) [58,59,83]. Mineral-ion wasting and osteopenia in humans harboring NHERF1 mutations underscores the importance of this interaction [25,26]. The published studies disclosed that the binding of the C-terminal motif of PTHR to NHERF1 involves regions outside the canonical core-binding PDZ boundaries [35]. The molecular determinants beyond the canonical binding site disclose a distinct electrostatic network playing a specific role in recognizing the PTHR C-terminus by the PDZ domains of SNX27 [84] and Scribble [85]. Currently, there is no information about the exact site of NHERF1 modification or the effect of phosphorylation by PKA. Several studies focused on NHERF1 post-translational modification by PKC [53,86–88], Akt [89], and Cdc2 [90]. PKC-induced Ser^77^/Thr^95^ phosphorylation in PDZ1 uncouples NPT2A and terminates PTH-sensitive phosphate transport [91,92]. Less is known about the physiological importance of Ser^339^ and Ser^340^, NHERF1 phosphorylation sites located in the flexible linker between PDZ2 and the EBD. It was shown that the phosphorylation-mimicking NHERF1 S^339^D/S^340^D mutant has a better binding affinity and stoichiometry for the carboxy-terminal PDZ-binding ligand of CFTR [88]. These results let us hypothesize that in the cellular environment, phosphorylation of these residues by PKC may promote conformational changes in NHERF1 or disrupt the autoinhibition interaction between NHERF1 PDZ2 and its own the carboxy-terminal PDZ ligand or both, making the PDZ2 domain more available for cellular targets including kinases, receptors, and signaling proteins [88]. How NHERF1 conformational rearrangement and plasticity regulate NHERF1-dependent hormone-regulated phosphate transport remains to be investigated.

## GRK6A

G protein-coupled receptor kinase 6A (GRK6A) constitutively or in response to PTH phosphorylates NHERF1 Ser^290^ [16,17]. The phosphorylation/dephosphorylation cycle at NHERF1 Ser^290^ modulates NPT2A-dependent phosphate uptake through a reversible NHERF1-NPT2A association-dissociation mechanism [93]. GRK6A, like NPT2A, possesses a canonical Class I type PDZ ligand motif (-Thr^−2^Arg^−1^Leu^0^) at its C-terminus. While NHERF1 PDZ1 binds GRK6A with high affinity, the weak interaction between PDZ2 and GRK6A promotes NHERF1 Ser^290^ phosphorylation [16], required for hormone-mediated inhibition of phosphate transport [17]. The interaction mechanism between PDZ2 and GRK6A may involve phospho-Ser^162^ in PDZ2, a PKCα site [53]. Our work [17,18] and that of others established that PKCα mediates PTH actions. Hence, PTH action entails phosphorylation of NHERF1 Ser^162^ by PKCα. The latter promotes conformational changes in NHERF1 and increases the binding affinity between PDZ2 and GRK6A [18]. Whether the interaction between PDZ2 and the PKCα C-terminal PDZ-ligand (-Ser^−2^Ala^−1^V^0(672)^) is necessary for phosphorylation of Ser^162^ remains to be established.

## Kinases (with PDZ ligands) involved in FGF23-regulated phosphate transport

**Figure 5:**
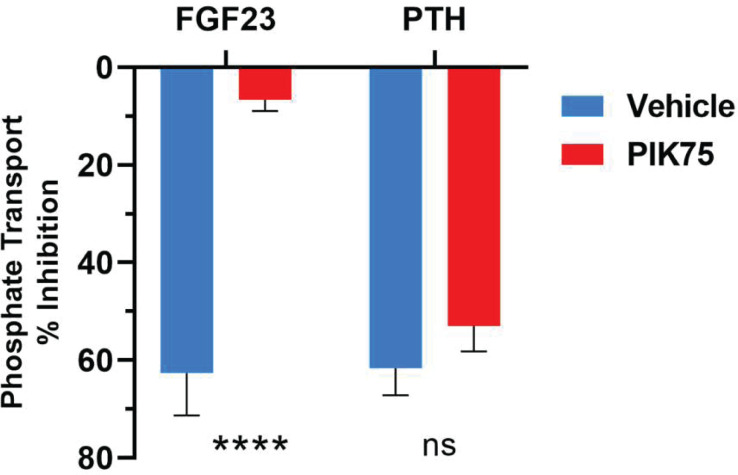
Effect of PIK75 inhibitor on FGF23- and PTH-sensitive phosphate transport PIK75, p38γ inhibitor, blocks FGF23 but not PTH inhibition of phosphate transport. OK cells were treated with 100 nM FGF23 or PTH for 2 hr in the absence or presence of PIK75 (10 μM). Phosphate transport was measured as ^32^P phosphate uptake, as described in detail [33]. Results report the mean ± SD (*n*=3, ****P<0.0001, ANOVA).

NHERF1 residues phosphorylated by FGF23 have not been described, though FGF23 clearly leads to NHERF1 phosphorylation [13,63]. We and others showed that ERK1/2 and SGK1 are downstream modulators of FGF23 signaling [11,36]. In agreement with previous studies, the ERK inhibitor, PD98059, blocked PTH and FGF23 actions on phosphate transport. The MAP kinase inhibitor, SB203580, only interfered with FGF23. The JNK inhibitor, SP600125, did not affect phosphate uptake [36]. Thus, ERK1/2 participates in mutual PTH/FGF23 regulation. PIK75, a p38γ-specific inhibitor [94], blocked FGF23 action without affecting PTH, as shown by pilot studies (Figure 5). Thus, p38γ may be a new mediator of FGF23-regulated phosphate transport. Similar to GRK6A [18], SGK1 and p38γ MAP kinase have Class I type PDZ-binding motifs at their C-termini, may interact with NHERF1 PDZ domains, and phosphorylate NHERF1 controlling association and disassembly of the NHERF1–NPT2A complex, NPT2A endocytosis and cessation of phosphate transport.

### *p38γ* (MAPK12)

**Figure 6:**
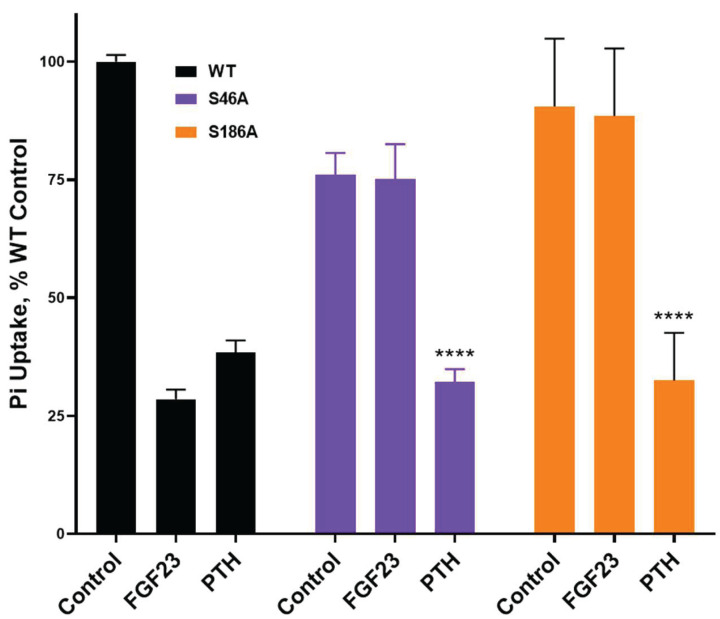
Effect of the serine/alanine replacement in NHERF1 on FGF23- and PTH-sensitive phosphate transport NHERF1 Ser^46^ and Ser^186^ are predicted MAP kinase phosphorylation sites. NHERF1 Ser^46^Ala and Ser^186^Ala substitution selectively abolish FGF23-inhibited phosphate transport at comparable transfection efficiency. OKH cells were transiently transfected with WT-NHERF1 or NHERF1 Ser^46^Ala or Ser^186^Ala variants. Cells were treated with vehicle or 100 nM PTH(1-34) or FGF23. NHERF1 constructs were prepared as before [18]. Phosphate transport was measured as ^32^P phosphate uptake, as described [33]. Results report the mean ± SD (*n*=4, ****P<0.0001, ANOVA).

p38γ, unlike p38α, p38β, or p38δ isoforms, uniquely possesses a canonical C-terminal PDZ ligand (-Glu^−3^Thr^−2^Pro^−1^Leu^0(367)^). p38γ forms multiprotein complexes with PDZ domain-containing proteins and phosphatases [95,96]. This raises the hypothesis that NHERF1 is a p38γ substrate and that p38γ C-terminal PDZ-ligand binding to NHERF1 promotes NHERF1 phosphorylation required for FGF23-sensitive phosphate transport. Although the p38γ C-terminal sequence (-Glu^−3^Thr^−2^Pro^−1^Leu^0^) is permissive for binding NHERF1 PDZ1 or PDZ2, we consider PDZ2 as a regulatory domain involved in the interactions with different targets, including p38γ [18], whereas PDZ1 interacts with NPT2A and defines basic phosphate transport [33]. NHERF1 has four *in silico* predicted MAP kinase consensus sites [97,98]. ^44^PGSP^47^ and ^184^PDSP^187^ sites are in PDZ1 and PDZ2, respectively, and ^280^SP^281^ and ^302^SP^303^ are located in the NHERF1 flexible linker between PDZ2 and EBD. Substitution of Ser^46^ or Ser^186^ by Ala eliminates FGF23-sensitive phosphate uptake without affecting PTH action, as shown by preliminary results (Figure 6), consistent with the idea that the p38γ MAP kinase is involved in FGF23 action on NPT2A-mediated phosphate transport. Phosphoresistant NHERF1 Ser^280^Ala or Ser^302^Ala replacements do not interfere with PTH action on phosphate transport and behave as WT NHERF1 [17], thereby suggesting that Ser^280^ and Ser^302^ are not involved in PTH-induced phosphorylation. We cannot exclude the possibility that the FGF23-p38γ or FGF23-ERK1/2 pathway impinges on the phosphorylation of Ser^280^ and Ser^302^. Whether Ser^46^, Ser^186^, Ser^280^, and Ser^302^ are p38γ or ERK1/2 phosphorylation sites contributing to FGF23-stimulated phosphorylation remains to be confirmed.

### PIN1

ERKs and p38 MAP kinase are proline-directed kinases (phospho-Ser/Thr-Pro). Proline uniquely adopts *cis* and *trans* conformations catalyzed by peptidyl-prolyl isomerases (PPIases) [99]. NHERF1 has four ‘SerPro’ sequences. Peptidyl-prolyl isomerases PIN1 is associated with phosphorylated NHERF1. Dephosphorylation of NHERF1 was blocked in the presence of juglone, a PIN1-selective inhibitor. This study demonstrates that PIN1 regulates phosphorylation-dephosphorylation of NHERF1 [90]. Because phosphorylation-dephosphorylation cycling is necessary for hormone-mediated phosphate transport [17], we speculate that PIN1 may be an unrecognized regulator of hormone-mediated inhibition of phosphate uptake. To test this idea, we measured NPT2A-dependent hormone-sensitive phosphate transport in OK cells treated with FGF23 [33] in the presence of juglone, rapamycin, and FK506. The pilot observations demonstrate that FGF23- and PTH-sensitive phosphate uptake was terminated in the presence of PIN1 inhibitors. These data corroborate our previous observation that ERK1/2 is involved in FGF23 and PTH action on phosphate transport [36].

### SGK1

SGK1 (serum and glucocorticoid-regulated kinase 1) belongs to the family of SGK kinase proteins [100]. SGK1 is a downstream modulator of FGF23-sensitive phosphate transport [36] and may interact with NHERF1 [101]. SGK1 carries a canonical Class I type PDZ-binding motif (-Asp^−3^Ser^−2^Phe^−1^Leu^0^) at its C-terminus. SGK1 binds and phosphorylates NHERF2 PDZ1 [102,103]. NHERF2, like NHERF1, contains two PDZ domains and an ezrin-binding domain at its C-terminus. NHERF1 PDZ2 shares high structural and sequence homology to NHERF2 PDZ1. Thus, NHERF1 binding of SGK1 via PDZ2 is predicted. NHERF1 has a putative SGK1 phosphorylation consensus sequence ^151^Arg-Pro-Arg-Leu-Cys-**Thr^156^**-Met [R-X-R-X-X-(S/T)-phi (X = any amino acid, R = arginine, S = serine, T = threonine, phi = hydrophobic amino acid) [100,104]. SGK1 likely phosphorylates NHERF1, although Thr^156^ was characterized as an AKT1 phosphorylation site [89]. Unlike SGK1, which binds NHERF1, AKT1 lacks a PDZ ligand. SGK1 may act at a non-canonical NHERF1 site such as Ser^340^. Alternatively, SGK1 could phosphorylate and activate ERK1/2, p38γ, or phosphorylate an unidentified NHERF1-interacting protein. It is essential to uncover how SGK1 affects the NHERF1-NPT2A complex and NPT2A localization and determine its upstream and downstream partners in FGF23 action on phosphate transport.

## Phosphatases

Reversible NHERF1 Ser^290^ phosphorylation-dephosphorylation regulates NPT2A-NHERF1 complex turnover and FGF23- and PTH-triggered inhibition of phosphate transport. Protein phosphatase 1α (PP1α) dephosphorylates phospho-Ser^290^ following binding to a conserved NHERF1 ^257^VPF^259^ (VxF/W) PP1 motif [105]. Mutating ^257^VPF^259^ eliminated PP1 binding and blunted dephosphorylation. Tautomycin, a specific PP1 inhibitor [106], blocked PP1 activity and abrogated NPT2A-dependent PTH-sensitive phosphate transport [105].

DUSP10 is a dual-specificity p38 phosphatase [107] that specifically dephosphorylates threonine/serine and tyrosine residues of the Ser/Thr-X-Tyr motif within p38. Moreover, it harbors a canonical Class I type C-terminal PDZ ligand (-Glu^−3^Thr^−2^Val^−1^Val^0^), suggesting binding to NHERF1 PDZ2 and thus of particular interest. Whether DUSP10 regulates FGF23 action but not PTH should be confirmed using specific DUSP10 inhibitors.

## RGS14

**Figure 7:**
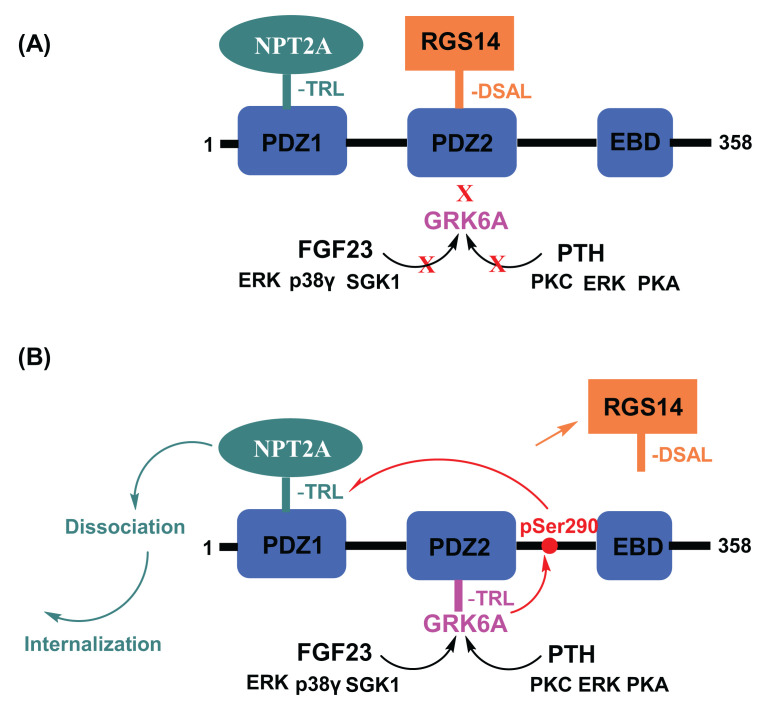
RGS14 is a novel regulator of hormone-sensitive phosphate transport (**A**) The C-terminal PDZ motif of human RGS14 binds NHERF1 PDZ2, stabilizes the NHERF1-NPT2A complex, and impedes the phosphaturic action of FGF23 and PTH. (**B**) Dissociation of RGS14 from NHERF1 restores FGF23- and PTH-stimulated phosphorylation of NHERF1, disassembly of the NHERF1-NPT2A complex, and FGF23- and PTH-mediated inhibition of phosphate uptake [110].

Regulators of G protein signaling (RGSs) are GTPase-activating proteins that accelerate GTP hydrolysis and terminate GPCR signaling [108–110]. RGS14 is an unusual multifunctional scaffolding protein that integrates G protein, mitogen-activated protein kinase, and Ca^2+^/calmodulin signaling pathways [111,112]. The best evidence for the activities of Rgs14 comes from studies conducted on rodent brains, where it tonically suppresses hippocampal-based learning and synaptic plasticity [111, 112, 114], in the heart, where it reduces myocardial remodeling [115], and in brown adipose tissue metabolism associated with longevity [116]. Information regarding human RGS14 is far less. Two tandem Ras/Rap-binding domains that bind active H-Ras and Rap2 [118], an amino-terminal RGS domain that binds Gαi/o-GTP and functions as a GTPase-activating protein to limit G protein signaling [113, 117], and a G protein regulator (also known as GoLoco) motif that binds inactive Gαi1/3 to anchor Rgs14 at membranes [119] are among the common domain structures shared by human and rodent Rgs14. Human, primate, and ovine RGS14 differ from the rodent protein because they contain a C-terminal Class I type PDZ-recognition sequence (-Asp^−3^Ser^−2^Ala^−1^Leu^0(566)^) [110]. The difference arises from a UAG stop codon in exon 15 of all species other than primates on sheep terminating mRNA translation. The corresponding primate codon is CAG, which encodes the Gln (Q) at RGS14 546 [120]. The potential interaction and functional consequences of RGS14 engagement with PDZ proteins have not been described. The two NHERF1 PDZ domains share identical GYGF core-binding motifs, but RGS14 selectively binds PDZ2 in cells [110] (Figure 7). Proximity-ligation analysis (PLA) at constitutive expression levels in human proximal convoluted tubule (HPCT) cells demonstrated the presence and significant colocalization of RGS14 with NHERF1. More importantly, RGS14 does not interfere with the interaction between NPT2A and NHERF1 PDZ1 (Figure 7) required for hormone-triggered inhibition of phosphate uptake. PTH failed to affect phosphate transport in cells expressing RGS14, suggesting it tonically suppresses PTH-sensitive but not basal phosphate uptake [110]. We expect comparable results for FGF23 action. These findings indicate that RGS14 is a novel regulator of hormone-sensitive phosphate transport. Additional studies are required to understand how RGS14 abundance may contribute to hormone resistance and hyperphosphatemia.

RGS12 is a PDZ-containing protein with a C-terminal Class I type PDZ-ligand motif (Thr^−2^Phe^−1^Val^0^). We predict that RGS12 with an intact carboxy terminus (residues 667-1447) will block FGF23- and PTH-sensitive phosphate transport. Further studies may shed light on the role of the RGS12 C-terminal PDZ ligand and PDZ domain on NPT2A-dependent hormone-regulated phosphate homeostasis.

## Concluding remarks

PTHR and FGFR1, two structurally distinct transmembrane receptors, regulate NPT2A-dependent phosphate homeostasis. Both enable signaling pathways converging on the NPT2A-NHERF1 complex. Mutations in NPT2A or NHERF1 cause elevated renal phosphate excretion and hypophosphatemia in patients, thus highlighting an essential role of the NPT2A-NHERF1 axis in bone and kidney physiology. NHERF1 PDZ1 domain determines the NPT2A apical membrane localization and basal phosphate transport. PDZ2 tethers PDZ and non-PDZ cellular targets, including kinases involved in FGF23 and PTH signaling, and serves as a regulatory domain. Signaling cascades initiated by PTH and FGF23 and controlled NPT2A and NHERF1 phosphorylation are required for hormone-sensitive phosphate transport in health and disease. Identifying FGF23 and PTH downstream regulators and mechanisms underlying hormone-induced phosphorylation will be critical for understanding disordered renal phosphate transport and mineral-ion metabolism associated with chronic kidney disease-mineral and bone disorder (CKD-MBD) and related phosphate-wasting disorders.

## Abbreviations

CKD: chronic kidney disease
FGF23: fibroblast growth factor-23
GRK6A: G protein-coupled receptor kinase 6A
HPCT: human proximal convoluted tubule
OK cells: opossum kidney cells
OKH cells: NHERF1-deficient opossum kidney cells
MBD: mineral and bone disorder
PLA: proximity-ligation analysis
PTH: parathyroid hormone
RGS: regulator of G protein signaling

## References

[1] Drueke T.B. (2010) Klotho, FGF23, and FGF receptors in chronic kidney disease: a yin-yang situation? Kidney Int. 78, 1057–1060 10.1038/ki.2010.339 21076444

[2] Gutierrez O.M. (2015) Contextual poverty, nutrition, and chronic kidney disease. Adv. Chronic Kidney Dis. 22, 31–38 10.1053/j.ackd.2014.05.005 25573510 PMC4291540

[3] Cannata-Andia J.B., Martin-Carro B., Martin-Virgala J., Rodriguez-Carrio J., Bande-Fernandez J.J., Alonso-Montes C. et al. (2021) Chronic kidney disease-mineral and bone disorders: pathogenesis and management. Calcif. Tissue Int. 108, 410–422 10.1007/s00223-020-00777-1 33190187

[4] Baum M. (2014) The bone kidney axis. Curr. Opin. Pediatr. 26, 177–179 10.1097/MOP.0000000000000071 24535498 PMC4074396

[5] Weinman E.J. and Lederer E.D. (2012) PTH-mediated inhibition of the renal transport of phosphate. Exp. Cell. Res. 318, 1027–1032 10.1016/j.yexcr.2012.02.037 22417892 PMC3334409

[6] Hernando N., Gisler S.M., Reining S.C., Deliot N., Capuano P., Biber J. et al. (2010) NaPi-IIa interacting proteins and regulation of renal reabsorption of phosphate. Urol. Res. 38, 271–276 10.1007/s00240-010-0304-3 20665015

[7] Yamashita T., Konishi M., Miyake A., Inui K. and Itoh N. (2002) Fibroblast growth factor (FGF)-23 inhibits renal phosphate reabsorption by activation of the mitogen-activated protein kinase pathway. J. Biol. Chem. 277, 28265–28270 10.1074/jbc.M202527200 12032146

[8] Hollenstein K., de Graaf C., Bortolato A., Wang M.W., Marshall F.H. and Stevens R.C. (2014) Insights into the structure of class B GPCRs. Trends Pharmacol. Sci. 35, 12–22 10.1016/j.tips.2013.11.001 24359917 PMC3931419

[9] McGarvey J.C., Xiao K., Bowman S.L., Mamonova T., Zhang Q., Bisello A. et al. (2016) Actin-Sorting Nexin 27 (SNX27)-retromer complex mediates rapid parathyroid hormone receptor recycling. J. Biol. Chem. 291, 10986–11002 10.1074/jbc.M115.697045 27008860 PMC4900250

[10] Sutkeviciute I. and Vilardaga J.P. (2020) Structural insights into emergent signaling modes of G protein-coupled receptors. J. Biol. Chem. 295, 11626–11642 10.1074/jbc.REV120.009348 32571882 PMC7450137

[11] Erben R.G. (2016) Update on FGF23 and Klotho signaling. Mol. Cell. Endocrinol. 432, 56–65 10.1016/j.mce.2016.05.008 27178987

[12] McKenna M.J., Crowley R.K., Twomey P.J. and Kilbane M.T. (2021) Renal phosphate handling: independent effects of circulating FGF23, PTH, and calcium. JBMR Plus 5, e10437 10.1002/jbm4.10437 33615106 PMC7872336

[13] Andrukhova O., Streicher C., Zeitz U. and Erben R.G. (2016) Fgf23 and parathyroid hormone signaling interact in kidney and bone. Mol. Cell. Endocrinol. 436, 224–239 10.1016/j.mce.2016.07.035 27498418

[14] Ovejero D., Hartley I.R., de Castro Diaz L.F., Theng E., Li X., Gafni R.I. et al. (2021) PTH and FGF23 exert interdependent effects on renal phosphate handling: evidence from patients with hypoparathyroidism and hyperphosphatemic familial tumoral calcinosis treated with synthetic human PTH 1-34. J. Bone Miner. Res. 37, 179–184 34464000 10.1002/jbmr.4429 PMC13170488

[15] Bergwitz C. and Juppner H. (2010) Regulation of phosphate homeostasis by PTH, vitamin D, and FGF23. Annu. Rev. Med. 61, 91–104 10.1146/annurev.med.051308.111339 20059333 PMC4777331

[16] Hall R.A., Spurney R.F., Premont R.T., Rahman N., Blitzer J.T., Pitcher J.A. et al. (1999) G protein-coupled receptor kinase 6A phosphorylates the Na^(+)^/H^(+)^ exchanger regulatory factor via a PDZ domain-mediated interaction. J. Biol. Chem. 274, 24328–24334 10.1074/jbc.274.34.24328 10446210

[17] Zhang Q., Xiao K., Paredes J.M., Mamonova T., Sneddon W.B., Liu H. et al. (2019) Parathyroid hormone initiates dynamic NHERF1 phosphorylation cycling and conformational changes that regulate NPT2A-dependent phosphate transport. J. Biol. Chem. 294, 4546–4571 10.1074/jbc.RA119.007421 30696771 PMC6433080

[18] Vistrup-Parry M., Sneddon W.B., Bach S., Stromgaard K., Friedman P.A. and Mamonova T. (2021) Multisite NHERF1 phosphorylation controls GRK6A regulation of hormone-sensitive phosphate transport. J. Biol. Chem. 296, 100473 10.1016/j.jbc.2021.100473 33639163 PMC8042174

[19] Sneddon W.B., Syme C.A., Bisello A., Magyar C.E., Rochdi M.D., Parent J.L. et al. (2003) Activation-independent parathyroid hormone receptor internalization is regulated by NHERF1 (EBP50). J. Biol. Chem. 278, 43787–43796 10.1074/jbc.M306019200 12920119

[20] Wade J.B., Liu J., Coleman R.A., Cunningham R., Steplock D.A., Lee-Kwon W. et al. (2003) Localization and interaction of NHERF isoforms in the renal proximal tubule of the mouse. Am. J. Physiol. Cell Physiol. 285, C1494–C1503 10.1152/ajpcell.00092.2003 12917102

[21] Cheng S., Li Y., Yang Y., Feng D., Yang L., Ma Q. et al. (2013) Breast cancer-derived K172N, D301V mutations abolish Na^+^/H^+^ exchanger regulatory factor 1 inhibition of platelet-derived growth factor receptor signaling. FEBS Lett. 587, 3289–3295 10.1016/j.febslet.2013.08.026 24012959

[22] Shenolikar S., Voltz J.W., Minkoff C.M., Wade J.B. and Weinman E.J. (2002) Targeted disruption of the mouse NHERF-1 gene promotes internalization of proximal tubule sodium-phosphate cotransporter type IIa and renal phosphate wasting. Proc. Natl. Acad. Sci. U.S.A. 99, 11470–11475 10.1073/pnas.162232699 12169661 PMC123280

[23] Morales F.C., Takahashi Y., Kreimann E.L. and Georgescu M.M. (2004) Ezrin-radixin-moesin (ERM)-binding phosphoprotein 50 organizes ERM proteins at the apical membrane of polarized epithelia. Proc. Natl. Acad. Sci. U.S.A. 101, 17705–17710 10.1073/pnas.0407974101 15591354 PMC539771

[24] Weinman E.J., Mohanlal V., Stoycheff N., Wang F., Steplock D., Shenolikar S. et al. (2006) Longitudinal study of urinary excretion of phosphate, calcium, and uric acid in mutant NHERF-1 null mice. Am. J. Physiol. Renal. Physiol. 290, F838–F843 10.1152/ajprenal.00374.2005 16249272

[25] Karim Z., Gerard B., Bakouh N., Alili R., Leroy C., Beck L. et al. (2008) NHERF1 mutations and responsiveness of renal parathyroid hormone. N. Engl. J. Med. 359, 1128–1135 10.1056/NEJMoa0802836 18784102

[26] Courbebaisse M., Leroy C., Bakouh N., Salaun C., Beck L., Grandchamp B. et al. (2012) A new human NHERF1 mutation decreases renal phosphate transporter NPT2a expression by a PTH-independent mechanism. PLoS ONE 7, e34764 10.1371/journal.pone.0034764 22506049 PMC3323571

[27] Wang B., Means C.K., Yang Y., Mamonova T., Bisello A., Altschuler D.L. et al. (2012) Ezrin-anchored protein kinase A coordinates phosphorylation-dependent disassembly of a NHERF1 ternary complex to regulate hormone-sensitive phosphate transport. J. Biol. Chem. 287, 24148–24163 10.1074/jbc.M112.369405 22628548 PMC3397842

[28] Karthikeyan S., Leung T. and Ladias J.A. (2002) Structural determinants of the Na^+^/H^+^ exchanger regulatory factor interaction with the beta 2 adrenergic and platelet-derived growth factor receptors. J. Biol. Chem. 277, 18973–18978 10.1074/jbc.M201507200 11882663

[29] Mamonova T. and Friedman P.A. (2021) Noncanonical sequences involving NHERF1 interaction with NPT2A govern hormone-regulated phosphate transport: binding outside the box. Int. J. Mol. Sci. 22, 15 10.3390/ijms22031087 PMC7866199 33499384

[30] Mahon M.J. (2009) The parathyroid hormone 1 receptor directly binds to the FERM domain of ezrin, an interaction that supports apical receptor localization and signaling in LLC-PK1 cells. Mol. Endocrinol. 23, 1691–1701 10.1210/me.2009-0164 19608645 PMC2754900

[31] Cunningham R., E X., Steplock D., Shenolikar S. and Weinman E.J. (2005) Defective PTH regulation of sodium-dependent phosphate transport in NHERF-1^−^/^-^ renal proximal tubule cells and wild-type cells adapted to low-phosphate media. Am. J. Physiol. Renal. Physiol. 289, F933–F938 10.1152/ajprenal.00005.2005 15942053

[32] Mamonova T., Zhang Q., Khajeh J.A., Bu Z., Bisello A. and Friedman P.A. (2015) Canonical and noncanonical sites determine NPT2A binding selectivity to NHERF1 PDZ1. PloS ONE 10, e0129554 10.1371/journal.pone.0129554 26070212 PMC4466390

[33] Sneddon W.B., Friedman P.A. and Mamonova T. (2023) Mutations in an unrecognized internal NPT2A PDZ motif disrupt phosphate transport and cause congenital hypophosphatemia. Biochem. J. 480, 685–699 10.1042/BCJ20230020 37132631 PMC10442799

[34] Mamonova T., Kurnikova M. and Friedman P.A. (2012) Structural basis for NHERF1 PDZ domain binding. Biochemistry 51, 3110–3120 10.1021/bi201213w 22429102 PMC3323774

[35] Mamonova T., Zhang Q., Chandra M., Collins B.M., Sarfo E., Bu Z. et al. (2017) Origins of PDZ binding specificity. A computational and experimental study using NHERF1 and the parathyroid hormone receptor. Biochemistry 56, 2584–2593 10.1021/acs.biochem.7b00078 28376304 PMC5479578

[36] Sneddon W.B., Ruiz G.W., Gallo L.I., Xiao K., Zhang Q., Rbaibi Y. et al. (2016) Convergent signaling pathways regulate parathyroid hormone and fibroblast growth factor-23 action on NPT2A-mediated phosphate transport. J. Biol. Chem. 291, 18632–18642 10.1074/jbc.M116.744052 27432882 PMC5009241

[37] Sasaki S., Shiozaki Y., Hanazaki A., Koike M., Tanifuji K., Uga M. et al. (2022) Tmem174, a regulator of phosphate transporter prevents hyperphosphatemia. Sci. Rep. 12, 6353 10.1038/s41598-022-10409-3 35428804 PMC9012787

[38] Miyazaki-Anzai S., Keenan A.L., Blaine J. and Miyazaki M. (2022) Targeted disruption of a proximal tubule-specific TMEM174 gene in mice causes hyperphosphatemia and vascular calcification. J. Am. Soc. Nephrol. 33, 1477–1486 10.1681/ASN.2021121578 35459732 PMC9342641

[39] Tomoe Y., Segawa H., Shiozawa K., Kaneko I., Tominaga R., Hanabusa E. et al. (2010) Phosphaturic action of fibroblast growth factor 23 in Npt2 null mice. Am. J. Physiol. Renal. Physiol. 298, F1341–F1350 10.1152/ajprenal.00375.2009 20357029

[40] Levi M. and Gratton E. (2019) Visualizing the regulation of SLC34 proteins et the apical membrane. Pflugers Arch. 471, 533–542 10.1007/s00424-018-02249-w 30613865 PMC6436987

[41] Penido M.G. and Alon U.S. (2012) Phosphate homeostasis and its role in bone health. Pediatr. Nephrol. 27, 2039–2048 10.1007/s00467-012-2175-z 22552885 PMC3461213

[42] Hernando N., Deliot N., Gisler S.M., Lederer E., Weinman E.J., Biber J. et al. (2002) PDZ-domain interactions and apical expression of type IIa Na/P_i_ cotransporters. Proc. Natl. Acad. Sci. U.S.A. 99, 11957–11962 10.1073/pnas.182412699 12192091 PMC129376

[43] Weinman E.J., Steplock D., Shenolikar S. and Biswas R. (2011) Fibroblast growth factor-23-mediated inhibition of renal phosphate transport in mice requires sodium-hydrogen exchanger regulatory factor-1 (NHERF-1) and synergizes with parathyroid hormone. J. Biol. Chem. 286, 37216–37221 10.1074/jbc.M111.288357 21908609 PMC3199469

[44] Weinman E.J., Steplock D., Shenolikar S. and Blanpied T.A. (2011) Dynamics of PTH-induced disassembly of Npt2a/NHERF-1 complexes in living OK cells. Am. J. Physiol. Renal. Physiol. 300, F231–F235 10.1152/ajprenal.00532.2010 21048030 PMC3023216

[45] Ardura J.A. and Friedman P.A. (2011) Regulation of G protein-coupled receptor function by Na^+^/H^+^ exchange regulatory factors. Pharmacol. Rev. 63, 882–900 10.1124/pr.110.004176 21873413 PMC3186079

[46] Wang B., Bisello A., Yang Y., Romero G.G. and Friedman P.A. (2007) NHERF1 regulates parathyroid hormone receptor membrane retention without affecting recycling. J. Biol. Chem. 282, 36214–36222 10.1074/jbc.M707263200 17884816

[47] Amizuka N., Lee H.S., Kwan M.Y., Arazani A., Warshawsky H., Hendy G.N. et al. (1997) Cell-specific expression of the parathyroid hormone (PTH)/PTH-related peptide receptor gene in kidney from kidney-specific and ubiquitous promoters. Endocrinology 138, 469–481 10.1210/endo.138.1.4845 8977437

[48] Ba J., Brown D. and Friedman P.A. (2003) Calcium-sensing receptor regulation of PTH-inhibitable proximal tubule phosphate transport. Am. J. Physiol. Renal. Physiol. 285, F1233–F1243 10.1152/ajprenal.00249.2003 12952858

[49] Ardura J.A., Wang B., Watkins S.C., Vilardaga J.P. and Friedman P.A. (2011) Dynamic Na^+^/H^+^ exchanger regulatory factor-1 association and dissociation regulate parathyroid hormone receptor trafficking at membrane microdomains. J. Biol. Chem. 286, 35020–35029 10.1074/jbc.M111.264978 21832055 PMC3186428

[50] Wang B., Ardura J.A., Romero G., Yang Y., Hall R.A. and Friedman P.A. (2010) Na/H exchanger regulatory factors control parathyroid hormone receptor signaling by facilitating differential activation of G(alpha) protein subunits. J. Biol. Chem. 285, 26976–26986 10.1074/jbc.M110.147785 20562104 PMC2930697

[51] Ritter S.L. and Hall R.A. (2009) Fine-tuning of GPCR activity by receptor-interacting proteins. Nat. Rev. Mol. Cell Biol. 10, 819–830 10.1038/nrm2803 19935667 PMC2825052

[52] Salyer S., Lesousky N., Weinman E.J., Clark B.J., Lederer E.D. and Khundmiri S.J. (2011) Dopamine regulation of Na^+^-K^+^-ATPase requires the PDZ-2 domain of sodium hydrogen regulatory factor-1 (NHERF-1) in opossum kidney cells. Am. J. Physiol. Cell Physiol. 300, C425–C434 10.1152/ajpcell.00357.2010 21160026 PMC3063973

[53] Raghuram V., Hormuth H. and Foskett J.K. (2003) A kinase-regulated mechanism controls CFTR channel gating by disrupting bivalent PDZ domain interactions. Proc. Natl. Acad. Sci. U.S.A. 100, 9620–9625 10.1073/pnas.1633250100 12881487 PMC170967

[54] Bas D.C., Rogers D.M. and Jensen J.H. (2008) Very fast prediction and rationalization of pKa values for protein-ligand complexes. Proteins 73, 765–783 10.1002/prot.22102 18498103

[55] Lee-Kwon W., Kim J.H., Choi J.W., Kawano K., Cha B., Dartt D.A. et al. (2003) Ca2^+^-dependent inhibition of NHE3 requires PKC alpha which binds to E3KARP to decrease surface NHE3 containing plasma membrane complexes. Am. J. Physiol. Cell Physiol. 285, C1527–C1536 10.1152/ajpcell.00017.2003 12954600

[56] White A.D., Pena K.A., Clark L.J., Maria C.S., Liu S., Jean-Alphonse F.G. et al. (2021) Spatial bias in cAMP generation determines biological responses to PTH type 1 receptor activation. Sci Signal 14, eabc5944 10.1126/scisignal.abc5944 34609896 PMC8682804

[57] Pena K.A. (2022) Endosomal parathyroid hormone receptor signaling. Am. J. Physiol. Cell Physiol. 323, C783–C790 10.1152/ajpcell.00452.2021 35912987 PMC9467467

[58] Mahon M.J., Donowitz M., Yun C.C. and Segre G.V. (2002) Na^(+)^/H^(+)^ exchanger regulatory factor 2 directs parathyroid hormone 1 receptor signalling. Nature 417, 858–861 10.1038/nature00816 12075354

[59] Wheeler D., Garrido J.L., Bisello A., Kim Y.K., Friedman P.A. and Romero G. (2008) Regulation of parathyroid hormone type 1 receptor dynamics, traffic, and signaling by the Na^+^/H^+^ exchanger regulatory factor-1 in rat osteosarcoma ROS 17/2.8 cells. Mol. Endocrinol. 22, 1163–1170 10.1210/me.2007-0461 18202147 PMC2366176

[60] Gattineni J., Bates C., Twombley K., Dwarakanath V., Robinson M.L., Goetz R. et al. (2009) FGF23 decreases renal NaPi-2a and NaPi-2c expression and induces hypophosphatemia in vivo predominantly via FGF receptor 1. Am. J. Physiol. Renal. Physiol. 297, F282–F291 10.1152/ajprenal.90742.2008 19515808 PMC2724258

[61] Urakawa I., Yamazaki Y., Shimada T., Iijima K., Hasegawa H., Okawa K. et al. (2006) Klotho converts canonical FGF receptor into a specific receptor for FGF23. Nature 444, 770–774 10.1038/nature05315 17086194

[62] Kurosu H., Ogawa Y., Miyoshi M., Yamamoto M., Nandi A., Rosenblatt K.P. et al. (2006) Regulation of fibroblast growth factor-23 signaling by klotho. J. Biol. Chem. 281, 6120–6123 10.1074/jbc.C500457200 16436388 PMC2637204

[63] Andrukhova O., Zeitz U., Goetz R., Mohammadi M., Lanske B. and Erben R.G. (2012) FGF23 acts directly on renal proximal tubules to induce phosphaturia through activation of the ERK1/2-SGK1 signaling pathway. Bone 51, 621–628 10.1016/j.bone.2012.05.015 22647968 PMC3419258

[64] Hernando N., Gagnon K. and Lederer E. (2021) Phosphate transport in epithelial and nonepithelial tissue. Physiol. Rev. 101, 1–35 10.1152/physrev.00008.2019 32353243

[65] Lederer E. (2014) Renal phosphate transporters. Curr. Opin. Nephrol. Hypertens. 23, 502–506 10.1097/MNH.0000000000000053 25028980 PMC4361807

[66] Biber J., Custer M., Kaissling B., Lötscher M. and Murer H. (1993) Molecular localization of Na/P_i_-cotransport (NaPi-2) in the nephron of rat kidney. J. Am. Soc. Nephrol. 4, 703

[67] Beck L., Karaplis A.C., Amizuka N., Hewson A.S., Ozawa H. and Tenenhouse H.S. (1998) Targeted inactivation of Npt2 in mice leads to severe renal phosphate wasting, hypercalciuria, and skeletal abnormalities. Proc. Natl. Acad. Sci. U.S.A. 95, 5372–5377 10.1073/pnas.95.9.5372 9560283 PMC20268

[68] Lundquist P., Murer H. and Biber J. (2007) Type II Na^+^-Pi cotransporters in osteoblast mineral formation: regulation by inorganic phosphate. Cell. Physiol. Biochem. 19, 43–56 10.1159/000099191 17310099

[69] Takashi Y., Sawatsubashi S., Endo I., Ohnishi Y., Abe M., Matsuhisa M. et al. (2021) Skeletal FGFR1 signaling is necessary for regulation of serum phosphate level by FGF23 and normal life span. Biochem. Biophys. Rep. 27, 101107 10.1016/j.bbrep.2021.101107 34458594 PMC8379418

[70] Fenollar-Ferrer C., Forster I.C., Patti M., Knoepfel T., Werner A. and Forrest L.R. (2015) Identification of the first sodium binding site of the phosphate cotransporter NaPi-IIa (SLC34A1). Biophys. J. 108, 2465–2480 10.1016/j.bpj.2015.03.054 25992725 PMC4457043

[71] Fenollar-Ferrer C. and Forrest L.R. (2019) Structural models of the NaPi-II sodium-phosphate cotransporters. Pflugers Arch. 471, 43–52 10.1007/s00424-018-2197-x 30175376 PMC6325988

[72] Jumper J., Evans R., Pritzel A., Green T., Figurnov M., Ronneberger O. et al. (2021) Highly accurate protein structure prediction with AlphaFold. Nature 596, 583–589 10.1038/s41586-021-03819-2 34265844 PMC8371605

[73] Karim-Jimenez Z., Hernando N., Biber J. and Murer H. (2000) A dibasic motif involved in parathyroid hormone-induced down-regulation of the type IIa NaPi cotransporter. Proc. Natl. Acad. Sci. U.S.A. 97, 12896–12901 10.1073/pnas.220394197 11050158 PMC18861

[74] Hillier B.J., Christopherson K.S., Prehoda K.E., Bredt D.S. and Lim W.A. (1999) Unexpected modes of PDZ domain scaffolding revealed by structure of nNOS-syntrophin complex. Science 284, 812–815 10.1126/science.284.5415.812 10221915

[75] Lazar C.S., Cresson C.M., Lauffenburger D.A. and Gill G.N. (2004) The Na^+^/H^+^ exchanger regulatory factor stabilizes epidermal growth factor receptors at the cell surface. Mol. Biol. Cell 15, 5470–5480 10.1091/mbc.e04-03-0239 15469991 PMC532026

[76] Rajagopal A., Braslavsky D., Lu J.T., Kleppe S., Clement F., Cassinelli H. et al. (2014) Exome sequencing identifies a novel homozygous mutation in the phosphate transporter SLC34A1 in hypophosphatemia and nephrocalcinosis. J. Clin. Endocrinol. Metab. 99, E2451–E2456 10.1210/jc.2014-1517 25050900 PMC4223446

[77] Kang S.J., Lee R. and Kim H.S. (2019) Infantile hypercalcemia with novel compound heterozygous mutation in SLC34A1 encoding renal sodium-phosphate cotransporter 2a: a case report. Ann. Pediatr. Endocrinol. Metab. 24, 64–67 10.6065/apem.2019.24.1.64 30943683 PMC6449619

[78] Ernst A., Appleton B.A., Ivarsson Y., Zhang Y., Gfeller D., Wiesmann C. et al. (2014) A structural portrait of the PDZ domain family. J. Mol. Biol. 426, 3509–3519 10.1016/j.jmb.2014.08.012 25158098

[79] Jankowski M., Hilfiker H., Biber J. and Murer H. (2001) The opossum kidney cell type IIa Na/P(i) cotransporter is a phosphoprotein. Kidney Blood Press. Res. 24, 1–4 10.1159/000054198 11173999

[80] Murer H. (2002) Functional domains in the renal type IIa Na/Pi-cotransporter. Kidney Int. 62, 375–382 10.1046/j.1523-1755.2002.00461.x 12109998

[81] Wang B., Yang Y., Abou-Samra A.B. and Friedman P.A. (2009) NHERF1 regulates parathyroid hormone receptor desensitization: interference with beta-arrestin binding. Mol. Pharmacol. 75, 1189–1197 10.1124/mol.108.054486 19188335 PMC2672812

[82] Wheeler D., Sneddon W.B., Wang B., Friedman P.A. and Romero G. (2007) NHERF-1 and the cytoskeleton regulate the traffic and membrane dynamics of G protein-coupled receptors. J. Biol. Chem. 282, 25076–25087 10.1074/jbc.M701544200 17599914

[83] Voltz J.W., Weinman E.J. and Shenolikar S. (2001) Expanding the role of NHERF, a PDZ-domain containing protein adapter, to growth regulation. Oncogene 20, 6309–6314 10.1038/sj.onc.1204774 11607833

[84] Clairfeuille T., Mas C., Chan A.S., Yang Z., Tello-Lafoz M., Chandra M. et al. (2016) A molecular code for endosomal recycling of phosphorylated cargos by the SNX27-retromer complex. Nat. Struct. Mol. Biol. 23, 921–932 10.1038/nsmb.3290 27595347

[85] Stewart B.Z., Mamonova T., Sneddon W.B., Javorsky A., Yang Y., Wang B. et al. (2023) Scribble scrambles parathyroid hormone receptor interactions to regulate phosphate and vitamin D homeostasis. Proc. Natl. Acad. Sci. U.S.A. 120, e2220851120 10.1073/pnas.2220851120 37252981 PMC10266016

[86] Fouassier L., Nichols M.T., Gidey E., McWilliams R.R., Robin H., Finnigan C. et al. (2005) Protein kinase C regulates the phosphorylation and oligomerization of ERM binding phosphoprotein 50. Exp. Cell. Res. 306, 264–273 10.1016/j.yexcr.2005.02.011 15878350

[87] Garbett D., LaLonde D.P. and Bretscher A. (2010) The scaffolding protein EBP50 regulates microvillar assembly in a phosphorylation-dependent manner. J. Cell Biol. 191, 397–413 10.1083/jcb.201004115 20937695 PMC2958488

[88] Li J., Poulikakos P.I., Dai Z., Testa J.R., Callaway D.J.E. and Bu Z. (2007) Protein kinase C phosphorylation disrupts Na^+^/H^+^ exchanger regulatory factor 1 autoinhibition and promotes cystic fibrosis transmembrane conductance regulator macromolecular assembly. J. Biol. Chem. 282, 27086–27099 10.1074/jbc.M702019200 17613530

[89] Song G.J., Leslie K.L., Barrick S., Mamonova T., Fitzpatrick J.M., Drombosky K.W. et al. (2015) Phosphorylation of ezrin-radixin-moesin-binding phosphoprotein 50 (EBP50) by Akt promotes stability and mitogenic function of S-phase kinase associated protein-2 (Skp2. J. Biol. Chem. 290, 2879–2887 10.1074/jbc.M114.609768 25492869 PMC4317011

[90] He J., Lau A.G., Yaffe M.B. and Hall R.A. (2001) Phosphorylation and cell cycle-dependent regulation of Na^+^/H^+^ exchanger regulatory factor-1 by Cdc2 kinase. J. Biol. Chem. 276, 41559–41565 10.1074/jbc.M106859200 11533036

[91] Voltz J.W., Brush M., Sikes S., Steplock D., Weinman E.J. and Shenolikar S. (2007) Phosphorylation of PDZ1 domain attenuates NHERF-1 binding to cellular targets. J. Biol. Chem. 282, 33879–33887 10.1074/jbc.M703481200 17895247

[92] Weinman E.J., Steplock D., Zhang Y., Biswas R., Bloch R.J. and Shenolikar S. (2010) Cooperativity between the phosphorylation of Thr^95^ and Ser^77^ of NHERF-1 in the hormonal regulation of renal phosphate transport. J. Biol. Chem. 285, 25134–25138 10.1074/jbc.M110.132423 20571032 PMC2919075

[93] Zhang Q., Xiao K., Paredas J.M., Mamonova T., Sneddon W.B., Liu H. et al. (2019) Parathyroid hormone initiates dynamic NHERF1 phosphorylation cycling and conformational changes that regulate NPT2A-dependent phosphate transport. J. Biol. Chem. 294, 4546–4571 10.1074/jbc.RA119.007421 30696771 PMC6433080

[94] Zhang X.H., Nam S., Wu J., Chen C.H., Liu X., Li H. et al. (2018) Multi-kinase inhibitor with anti-p38gamma activity in cutaneous T-Cell lymphoma. J. Invest. Dermatol. 138, 2377–2387 10.1016/j.jid.2018.04.030 29758280 PMC7269016

[95] Maisonneuve P., Caillet-Saguy C., Vaney M.C., Bibi-Zainab E., Sawyer K., Raynal B. et al. (2016) Molecular basis of the interaction of the human protein tyrosine phosphatase non-receptor type 4 (PTPN4) with the mitogen-activated protein kinase p38gamma. J. Biol. Chem. 291, 16699–16708 10.1074/jbc.M115.707208 27246854 PMC4974383

[96] Chen K.E., Lin S.Y., Wu M.J., Ho M.R., Santhanam A., Chou C.C. et al. (2014) Reciprocal allosteric regulation of p38gamma and PTPN3 involves a PDZ domain-modulated complex formation. Sci Signal 7, ra98 10.1126/scisignal.2005722 25314968

[97] Bardwell L. (2006) Mechanisms of MAPK signalling specificity. Biochem. Soc. Trans. 34, 837–841 10.1042/BST0340837 17052210 PMC3017501

[98] Pinna L.A. and Ruzzene M. (1996) How do protein kinases recognize their substrates? Biochim. Biophys. Acta 1314, 191–225 10.1016/S0167-4889(96)00083-3 8982275

[99] Zhou X.Z. and Lu K.P. (2016) The isomerase PIN1 controls numerous cancer-driving pathways and is a unique drug target. Nat. Rev. Cancer 16, 463–478 10.1038/nrc.2016.49 27256007

[100] Lang F., Bohmer C., Palmada M., Seebohm G., Strutz-Seebohm N. and Vallon V. (2006) (Patho)physiological significance of the serum- and glucocorticoid-inducible kinase isoforms. Physiol. Rev. 86, 1151–1178 10.1152/physrev.00050.2005 17015487

[101] Hall R.A. and Lefkowitz R.J. (2002) Regulation of G protein-coupled receptor signaling by scaffold proteins. Circ. Res. 91, 672–680 10.1161/01.RES.0000037000.74258.03 12386143

[102] Chun J., Kwon T., Lee E., Suh P.G., Choi E.J. and Sun Kang S. (2002) The Na^(+)^/H^(+)^ exchanger regulatory factor 2 mediates phosphorylation of serum- and glucocorticoid-induced protein kinase 1 by 3-phosphoinositide-dependent protein kinase 1. Biochem. Biophys. Res. Commun. 298, 207–215 10.1016/S0006-291X(02)02428-2 12387817

[103] Yun C.C., Palmada M., Embark H.M., Fedorenko O., Feng Y., Henke G. et al. (2002) The serum and glucocorticoid-inducible kinase SGK1 and the Na^+^/H^+^ exchange regulating factor NHERF2 synergize to stimulate the renal outer medullary K^+^ channel ROMK1. J. Am. Soc. Nephrol. 13, 2823–2830 10.1097/01.ASN.0000035085.54451.81 12444200

[104] Park J., Leong M.L., Buse P., Maiyar A.C., Firestone G.L. and Hemmings B.A. (1999) Serum and glucocorticoid-inducible kinase (SGK) is a target of the PI 3-kinase-stimulated signaling pathway. EMBO J. 18, 3024–3033 10.1093/emboj/18.11.3024 10357815 PMC1171384

[105] Liu H., Wang D., Zhang Q., Zhao Y., Mamonova T., Wang L. et al. (2019) Parallel posttranslational modification scanning enhancing hydrogen-deuterium exchange-mass spectrometry coverage of key structural regions. Anal. Chem. 91, 6976–6980 10.1021/acs.analchem.9b01410 31082219 PMC9270701

[106] Choy M.S., Swingle M., D'Arcy B., Abney K., Rusin S.F., Kettenbach A.N. et al. (2017) PP1:tautomycetin complex reveals a path toward the development of PP1-specific inhibitors. J. Am. Chem. Soc. 139, 17703–17706 10.1021/jacs.7b09368 29156132 PMC5729109

[107] Chen H.F., Chuang H.C. and Tan T.H. (2019) Regulation of dual-specificity phosphatase (DUSP) ubiquitination and protein stability. Int. J. Mol. Sci. 20, 1–17 10.3390/ijms20112668 PMC6600639 31151270

[108] Hollinger S. and Hepler J.R. (2002) Cellular regulation of RGS proteins: modulators and integrators of G protein signaling. Pharmacol. Rev. 54, 527–559 10.1124/pr.54.3.527 12223533

[109] Ross E.M. and Wilkie T.M. (2000) GTPase-activating proteins for heterotrimeric G proteins: regulators of G protein signaling (RGS) and RGS-like proteins. Annu. Rev. Biochem. 69, 795–827 10.1146/annurev.biochem.69.1.795 10966476

[110] Friedman P.A., Sneddon W.B., Mamonova T., Montanez-Miranda C., Ramineni S., Harbin N.H. et al. (2022) RGS14 regulates PTH- and FGF23-sensitive NPT2A-mediated renal phosphate uptake via binding to the NHERF1 scaffolding protein. J. Biol. Chem. 298, 101836 10.1016/j.jbc.2022.101836 35307350 PMC9035407

[111] Evans P.R., Dudek S.M. and Hepler J.R. (2015) Regulator of G protein signaling 14: a molecular brake on synaptic plasticity linked to learning and memory. Prog. Mol. Biol. Transl. Sci. 133, 169–206 10.1016/bs.pmbts.2015.03.006 26123307

[112] Evans P.R., Gerber K.J., Dammer E.B., Duong D.M., Goswami D., Lustberg D.J. et al. (2018) Interactome analysis reveals regulator of G protein signaling 14 (RGS14) is a novel calcium/calmodulin (Ca(^2+^)/CaM) and CaM kinase II (CaMKII) binding partner. J. Proteome Res. 17, 1700–1711 10.1021/acs.jproteome.8b00027 29518331 PMC6554723

[113] Hollinger S., Taylor J.B., Goldman E.H. and Hepler J.R. (2001) RGS14 is a bifunctional regulator of Galphai/o activity that exists in multiple populations in brain. J. Neurochem. 79, 941–949 10.1046/j.1471-4159.2001.00629.x 11739605

[114] Lee S.E., Simons S.B., Heldt S.A., Zhao M., Schroeder J.P., Vellano C.P. et al. (2010) RGS14 is a natural suppressor of both synaptic plasticity in CA2 neurons and hippocampal-based learning and memory. Proc. Natl. Acad. Sci. U.S.A. 107, 16994–16998 10.1073/pnas.1005362107 20837545 PMC2947872

[115] Li Y., Tang X.H., Li X.H., Dai H.J., Miao R.J., Cai J.J. et al. (2016) Regulator of G protein signalling 14 attenuates cardiac remodelling through the MEK-ERK1/2 signalling pathway. Basic Res. Cardiol. 111, 47 10.1007/s00395-016-0566-1 27298141 PMC4906057

[116] Vatner D.E., Zhang J., Oydanich M., Guers J., Katsyuba E., Yan L. et al. (2018) Enhanced longevity and metabolism by brown adipose tissue with disruption of the regulator of G protein signaling 14. Aging Cell 17, e12751 10.1111/acel.12751 29654651 PMC6052469

[117] Cho H., Kozasa T., Takekoshi K., De Gunzburg J. and Kehrl J.H. (2000) RGS14, a GTPase-activating protein for Gialpha, attenuates Gialpha- and G13alpha-mediated signaling pathways. Mol. Pharmacol. 58, 569–576 10.1124/mol.58.3.569 10953050

[118] Shu F.J., Ramineni S. and Hepler J.R. (2010) RGS14 is a multifunctional scaffold that integrates G protein and Ras/Raf MAPkinase signalling pathways. Cell. Signal. 22, 366–376 10.1016/j.cellsig.2009.10.005 19878719 PMC2795083

[119] Shu F.J., Ramineni S., Amyot W. and Hepler J.R. (2007) Selective interactions between Gi alpha1 and Gi alpha3 and the GoLoco/GPR domain of RGS14 influence its dynamic subcellular localization. Cell. Signal. 19, 163–176 10.1016/j.cellsig.2006.06.002 16870394

[120] Snow B.E., Antonio L., Suggs S., Gutstein H.B. and Siderovski D.P. (1997) Molecular cloning and expression analysis of rat Rgs12 and Rgs14. Biochem. Biophys. Res. Commun. 233, 770–777 10.1006/bbrc.1997.6537 9168931

